# Endogenous Energy Stores Maintain a High ATP Concentration for Hours in Glucose-Depleted Cultured Primary Rat Astrocytes

**DOI:** 10.1007/s11064-023-03903-1

**Published:** 2023-03-14

**Authors:** Antonia Regina Harders, Christian Arend, Sadhbh Cynth Denieffe, Julius Berger, Ralf Dringen

**Affiliations:** 1 Centre for Biomolecular Interactions Bremen Faculty 2 (Biology/Chemistry), University of Bremen, P.O. Box 330440, 28334 Bremen, Germany; 2 Centre for Environmental Research and Sustainable Technologies, University of Bremen, Bremen, Germany

**Keywords:** Astrocytes, ATP, Glucose, Fatty acids, Metabolism, Mitochondria

## Abstract

Adenosine triphosphate (ATP) is the central energy currency of all cells. Cultured primary rat astrocytes contain a specific cellular ATP content of 27.9 ± 4.7 nmol/mg. During incubation in a glucose- and amino acid-free incubation buffer, this high cellular ATP content was maintained for at least 6 h, while within 24 h the levels of ATP declined to around 30% of the initial value without compromising cell viability. In contrast, cells exposed to 1 mM and 5 mM glucose maintained the initial high cellular ATP content for 24 and 72 h, respectively. The loss in cellular ATP content observed during a 24 h glucose-deprivation was fully prevented by the presence of glucose, fructose or mannose as well as by the mitochondrial substrates lactate, pyruvate, β-hydroxybutyrate or acetate. The high initial specific ATP content in glucose-starved astrocytes, was almost completely abolished within 30 min after application of the respiratory chain inhibitor antimycin A or the mitochondrial uncoupler BAM-15, while these inhibitors lowered in glucose-fed cells the ATP content only to 60% (BAM-15) and 40% (antimycin A) within 5 h. Inhibition of the mitochondrial pyruvate carrier by UK5099 alone or of mitochondrial fatty acid uptake by etomoxir alone hardly affected the high ATP content of glucose-deprived astrocytes during an incubation for 8 h, while the co-application of both inhibitors depleted cellular ATP levels almost completely within 5 h. These data underline the importance of mitochondrial metabolism for the ATP regeneration of astrocytes and demonstrate that the mitochondrial oxidation of pyruvate and fatty acids strongly contributes to the maintenance of a high ATP concentration in glucose-deprived astrocytes.

## Introduction

The human brain is metabolically highly active. This organ uses around 20% of the glucose and oxygen that is consumed by the body [1, 2]. The extensive oxidative metabolism of the brain provides metabolic energy in form of ATP that serves mainly for fueling the transport processes involved in neuronal information transfer. Therefore, neurons are considered to consume most of the ATP that is generated in the brain [3]. In addition to neurons, the brain contains different types of glial cells in large numbers of which astrocytes are quantitatively the most important ones. Astrocytes are highly important partners of neurons in brain metabolism and homeostasis [1, 4–7]. To do so, astrocytes require also substantial amounts of ATP for their various functions and duties [1, 8], for example for neurotransmitter uptake [9], for maintaining the astrocytic membrane potential [10], for the glutamine synthetase-catalyzed amidation of glutamate to glutamine [11] and for storing substrates for energy production in the form of glycogen [12] and fatty acids [1, 13]. Therefore, the regeneration of ATP in astrocytes is crucial to maintain essential astrocytic functions for the brain.

ATP is generated in brain cells mainly by cytosolic glycolysis and by mitochondrial oxidative phosphorylation [14]. In culture, astrocytes rapidly convert large amounts of glucose to lactate with a ratio of more than 1.5 lactate released per glucose molecule consumed [15, 16], suggesting substantial ATP production in astrocytes via aerobic glycolysis [8]. This glycolytic phenotype of astrocytes is consistent with the observations that astrocytes survive an inactivated mitochondrial respiratory chain in culture and in vivo as purely glycolytic cells [16, 17]. However, astrocytes also possess an extensive mitochondrial metabolism [5], which is linked to efficient mitochondrial ATP regeneration. This is demonstrated by the rapid depletion of cellular ATP levels after application of inhibitors of mitochondrial oxidative phosphorylation to astrocytes that were co-exposed to a glycolysis inhibitor or that had been deprived of glucose [18–21]. Thus, despite the presence of many ATP consuming enzymes and transporters in astrocytes, a high cellular ATP level appears to be efficiently maintained by ATP regeneration via both cytosolic glycolysis and mitochondrial oxidative phosphorylation. Glucose is considered as the main physiological substrate of astrocytes [8]. However, at least in culture these cells are able to also utilize a substantial number of other substrates that can be oxidized to provide energy via mitochondrial processes, including pyruvate, lactate, acetate, ketone bodies as well as amino acids and fatty acids [1, 5, 21–25].

In order to investigate the potential of extracellular substrates and intracellular sources that contribute to the maintenance of a high astrocytic ATP concentration in astrocytes, we exposed cultured rat astrocytes to different substrates and metabolic inhibitors in the absence or the presence of glucose. Here we report that the high cellular ATP content of cultured astrocytes was fully maintained for many hours during incubation in the absence of glucose, whereas inhibition of mitochondrial phosphorylation or mitochondrial uncoupling depleted glucose-deprived astrocytes already within 30 min of their ATP. The data presented demonstrate that mitochondrial oxidative metabolism is essential to maintain high ATP levels in astrocytes, while accelerated glycolysis in glucose-treated astrocytes after inhibition of mitochondrial respiration can only partially maintain a high ATP content. In addition, the lowering of cellular ATP contents after application of inhibitors of mitochondrial transport processes suggests that mitochondrial oxidation of pyruvate and of fatty acids contribute to the maintenance of a high ATP concentration in starved astrocytes.

## Materials and Methods

### Materials

Fetal calf serum (FCS), antimycin A (A8674), and BAM-15 (SML1760) were purchased from Sigma-Aldrich (Steinheim, Germany; RRID:SCR_008988). Etomoxir (HY-50202) and UK5099 (HY-15475) were obtained from Merck (Darmstadt, Germany). Dulbecco’s modified Eagles medium (DMEM) and penicillin G/streptomycin sulfate solution were from Thermo Fisher Scientific (Schwerte, Germany). Acetate, bovine albumin, dimethyl sulfoxide (DMSO), perchloric acid, NAD^+^, NADH and NADP^+^ were from AppliChem (Darmstadt, Germany; RRID:SRC_005814). The Cell Titer Glo^®^ 2.0 ATP Assay Kit (G9241) was from Promega (Walldorf, Germany; RRID:SCR_006724). ATP and the enzymes used for the lactate and glucose assays were purchased at Roche Diagnostics (Mannheim, Germany; RRID:SCR_001326). All other basal chemicals were obtained from Sigma-Aldrich (Steinheim, Germany), Roth (Karlsruhe, Germany), Riedel-de Haën (Seelze, Germany) or Fluka (Buchs, Switzerland). Sterile cell culture materials as well as unsterile 96-well plates and black microtiter plates were obtained from Sarstedt (Nümbrecht, Germany).

### Astrocyte Cultures

Primary astrocyte cultures were prepared from the brains of newborn Wistar rats as previously described in detail [26]. The rats had been obtained from Charles River Laboratories (Sulzfeld, Germany; RRID:SCR_003792). Animals were treated in accordance to the State of Bremen, German and European animal welfare acts. The harvested cells were suspended in culture medium (90% DMEM containing 25 mM glucose, 44.6 mM sodium bicarbonate, 1 mM pyruvate, 20 U/mL penicillin G, 20 µg/mL streptomycin sulfate, supplemented with 10% FCS). One mL of the cell suspension containing 300,000 cells was seeded per well of 24-well dishes. The cells were cultured in a humidified atmosphere containing 10% CO_2_ in a Sanyo CO_2_ incubator (Osaka, Japan). The culture medium was renewed every seventh day and one day prior to experiments. For the current study, cultures of an age between 14 and 28 days were used. Astrocyte-rich primary cultures are strongly enriched in astrocytes (cells positive for glial fibrillary acidic protein) and contain only low numbers of other types of brain cells, including microglial cells, oligodendrocytes, and ependymal cells, but they do not contain neurons [15, 26, 27].

### Experimental Incubation of the Cells

All experiments were performed on astrocyte primary cultures in wells of 24-well dishes. The cells were washed twice with 1 mL pre-warmed (37 °C) glucose- and amino acid-free incubation buffer (IB; 145 mM NaCl, 20 mM HEPES, 5.4 mM KCl, 1.8 mM CaCl_2_, 1 mM MgCl_2_, 0.8 mM Na_2_HPO_4_, pH adjusted with NaOH to 7.4 at 37 °C). Incubations were carried out for the time periods indicated in the legends of the figures or the table at 37 °C in the humidified atmosphere of a CO_2_-free incubator in 250 µL glucose-free IB that had been supplemented with the given energy substrates and/or inhibitors of transporters or modulators of metabolic pathways. For substances that had been dissolved as stock solutions in DMSO, appropriate solvent controls containing the respective final concentration of DMSO (up to 1.1%) were performed. The presence of DMSO in a concentration of 1.1% during a 24 h incubation did not affect the cell viability nor the cellular ATP content compared with the respective control incubations in the absence of DMSO (data not shown). After the given incubation periods the incubation media were harvested for determination of extracellular lactate and glucose concentrations and extracellular lactate dehydrogenase (LDH) activity, while the cells were washed twice with 1 mL ice-cold (4 °C) phosphate-buffered saline (PBS; 10 mM potassium phosphate buffer pH 7.4 containing 150 mM NaCl) and lysed for ATP quantification.

### Determination of Cellular ATP Contents

Perchlorate lysates of cultured astrocytes were used to quantify cellular ATP contents as recently described [21] using a luciferine-luciferase-based [28] luminometric assay. Briefly, the cultures were washed twice with 1 mL ice-cold (4 °C) PBS and lysed in 200 µL of ice-cold 0.5 M HClO_4_ on ice for 1 min. The lysed cells were scraped off the dish with the help of a cell scraper and 10 µL of the collected lysates were diluted with 190 µL of 0.5 M HClO_4_ before the pH was neutralised by the addition of an appropriate amount of 2 M KOH. After 5 min of centrifugation at 12,100 x g the supernatant above the precipitated KClO_4_ was harvested and the pH adjusted to a neutral value by the addition of 10 µL of 1.4 M Tris/acetate buffer (pH 7.75). ATP standards in concentrations of up to 1000 nM in 0.5 M HClO_4_ were prepared and neutralised identically. Finally, 50 µL of neutralised lysate samples or ATP standards were diluted in wells of a black 96-well plate with 50 µL of the ATP detection reagent (Cell Titer Glo^®^ 2.0 ATP Assay Kit) to start the luciferase reaction. After 20 min of incubation, the luminescence signal was recorded by a Fluoroskan Ascent FL chemiluminescence plate reader (Thermo Fisher Scientific, Bremen, Germany). ATP values for the diluted cell samples were calculated by using the linear calibration curve generated from the values obtained for the ATP standards. Specific ATP contents were calculated by normalizing the ATP values determined to the initial cellular protein content of the cultures.

### Determination of Glucose and Lactate

The concentrations of extracellular glucose or lactate before and after a given incubation period were determined by coupled enzymatic assays in microtiter plate format as previously described in detail [26]. For analysis, media sample volumes of 10 or 20 µL were used. The assay used to quantify glucose is based on the phosphorylation of glucose by hexokinase to glucose-6-phosphate that is coupled to its subsequent oxidation by glucose-6-phosphate dehydrogenase. This oxidation generates NADPH in a concentration that is equimolar to that of the glucose present in the sample and is quantified by the absorption of NADPH at 340 nm [23]. For lactate quantification, the lactate in media samples is oxidized by LDH to pyruvate in a reaction that generates NADH which is also determined at 340 nm. Complete lactate oxidation requires coupling of the LDH reaction to that of glutamate-pyruvate transaminase that takes place in an alkaline glutamate buffer [23].

### Determination of Cell Viability and Protein Content

The extracellular activity of the cytosolic enzyme LDH was used to test for potential cell toxicity of a given experimental treatment. LDH activities in 10 µL media samples were compared with the initial cellular LDH activity to determine a percental toxicity as described previously in detail [26]. The initial cellular protein content per well was determined by the Lowry method [29] using bovine serum albumin as standard protein.

### Presentation of Data and Statistical Analysis

The data shown represent means ± standard deviation (SD) of values obtained from three or more experiments that were each performed in duplicates or triplicates on independently prepared astrocyte cultures. Analysis for statistical significance between groups of data was performed by ANOVA followed by the Bonferroni post-hoc test using the software GraphPad InStat 3. The calculated levels of significance compared to the indicated control conditions are given by *^, +^p < 0.05, **^, ++^p < 0.01 and ***^, +++^p < 0.001. p > 0.05 was considered as not significant.

## Results

### Specific Cellular ATP Content of Cultured Primary Rat Astrocytes

**Fig. 1:**
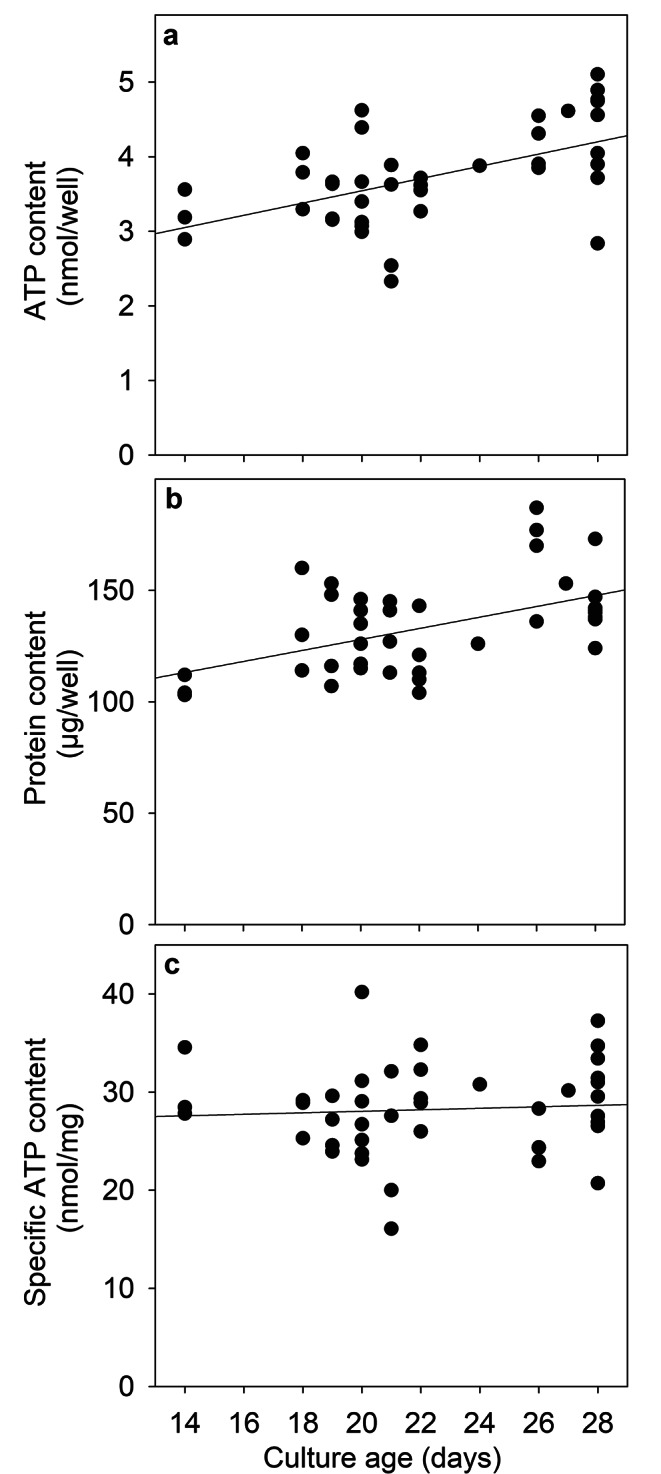
**Basal ATP content in untreated cultured astrocytes.** Astrocyte cultures were maintained in culture medium and analysed at the given day in culture for their ATP content (a) and protein content (b). The specific ATP content (c) was calculated by normalizing the ATP content per well (a) to the protein content of the respective culture (b). The data shown are mean values derived from a total of 42 experiments that had been performed on 27 independently prepared cultures

To determine the basal content of ATP in cultured astrocytes and to investigate a potential dependency of the ATP content on the age of the culture, basal ATP contents that had been determined in a total of 42 experiments (performed on 27 independently prepared cultures) were analysed (Fig. 1). With increasing culture age, the total ATP content per well (Fig. 1a) as well as the protein content per well (Fig. 1b) increased slightly, while the specific ATP content (nmol/mg protein) remained almost constant (Fig. 1c). The calculated average ATP content per well of untreated cultured astrocytes was 3.8 ± 0.6 nmol/well, the average protein content 135 ± 20 µg/well and the calculated specific ATP content was 27.9 ± 4.7 nmol/mg. For individual experiments substantial differences to the average specific ATP content of untreated cultures were observed, but no obvious age-dependent alterations of the specific ATP content of the cultures was found (Fig. 1c).

### Consequences of Glucose-Depletion on the Cellular ATP Content of Cultured Primary Astrocytes

**Fig. 2:**
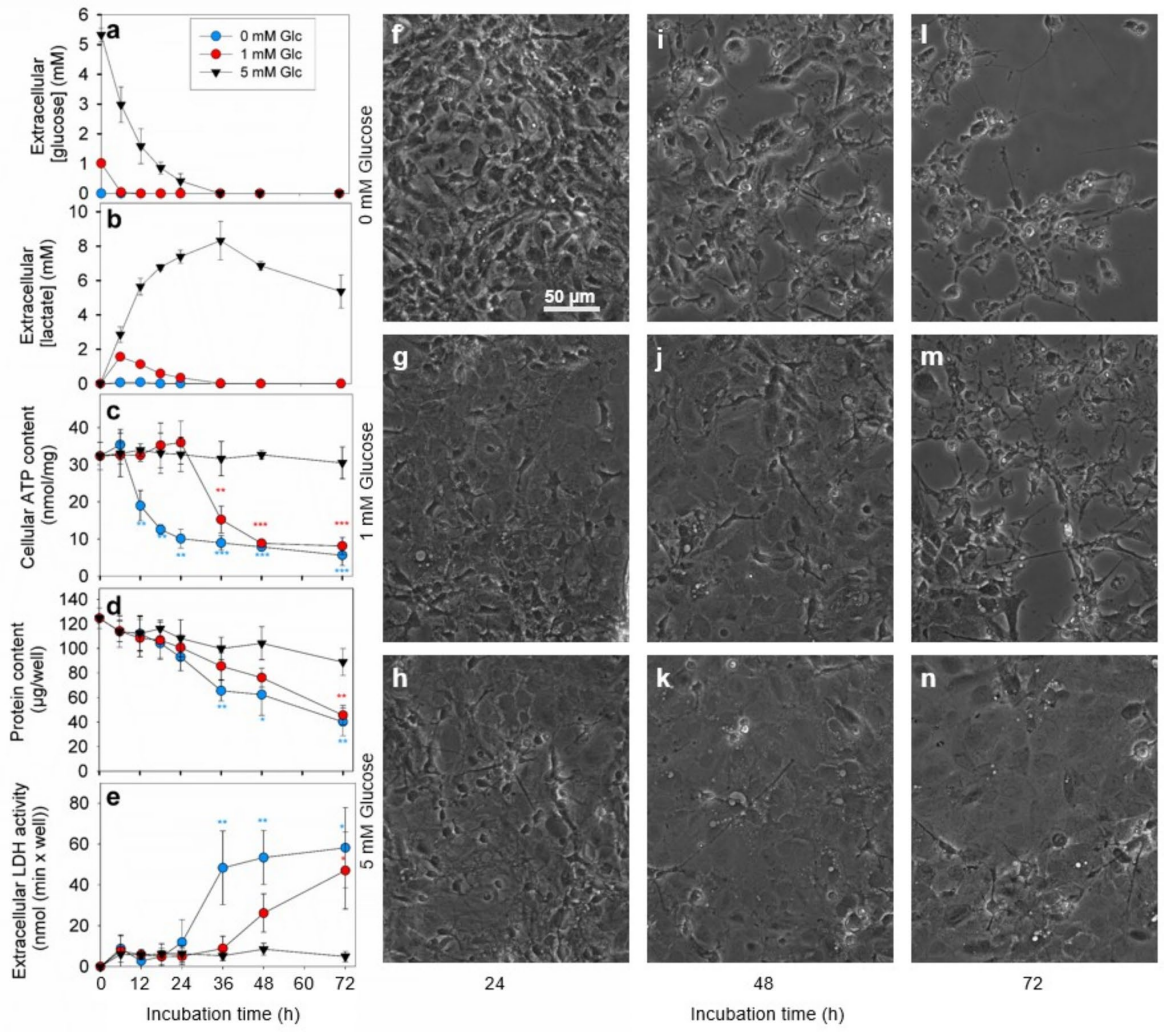
**Consequences of glucose deprivation in cultured astrocytes.** The cultures were incubated in an amino acid-free incubation buffer without (0 mM; a-f, i, l) or with 1 mM (a-e, g, j, m) or 5 mM (a-e, h, k, n) glucose for up to 3 days. After the indicated time points the extracellular concentrations of glucose (a) and lactate (b), the specific cellular ATP content (c), the cellular protein content (d), and the extracellular LDH activity (e) were determined and the cell morphology of the cultures (f-n) studied. At the onset of the incubation the cultures had an age between 18 and 20 days. The initial cellular LDH activity of the cultures was 113 ± 19 nmol/(min × well) and the initial protein content was 124 ± 8 µg/well. The data represent mean values ± SD of values obtained in experiments performed on 3 independently prepared cultures. In panels c-e, the significance of differences (ANOVA with Bonferroni post hoc test) compared to the data obtained for cells that had been exposed to 5 mM glucose are indicated in the colours of the respective symbols by * p < 0.05, ** p < 0.01 and *** p < 0.001. The size bar in panel f represents 50 μm and applies to panels f-n

To test for the consequences of glucose deprivation on the cellular ATP content, cultured astrocytes were incubated in the absence (0 mM) or the presence of 1 mM or 5 mM glucose in a basal sterile amino acid-free incubation buffer. For cells incubated in the absence of glucose, extracellular glucose was not detected, while cells exposed to 1 mM and 5 mM glucose had consumed the applied glucose completely within 6 and 36 h, respectively (Fig. 2a). As expected, only very low concentrations (up to 90 µM) of lactate were determined for glucose-starved cells, while the medium of astrocytes that had been exposed to 1 mM and 5 mM glucose contained maximal lactate concentrations of around 1.8 mM and 8 mM, respectively, after all the applied glucose had been metabolized by the cells (Fig. 2b). This extracellular lactate was subsequently consumed by the cells during longer incubations (Fig. 2b). Within the first 24 h of glucose deprivation the cell viability was not compromised for any of the conditions investigated as indicated by unaltered values for the cellular protein content (Fig. 2d) and by the low extracellular LDH activity detected (Fig. 2e).

The specific cellular ATP content of glucose-starved astrocytes remained high for at least 6 h, but gradually declined during longer incubations to around 30% of the initial value within 24 h of incubation (Fig. 2c). Longer incubations of starved astrocytes (no glucose initially applied) resulted in severe cell loss and toxicity detectable after 36 h of incubation as demonstrated by the significant decline in the cellular protein content (Fig. 2d), the strong increase in extracellular LDH activity (Fig. 2e) and a loss of cells visible in microscopic pictures of the cultures (Fig. 2i, l). For astrocytes that had been exposed to 1 mM glucose, the ATP content was maintained at the initial high levels for at least 24 h and gradually declined only during longer incubations to around 30% of the initial level (Fig. 2c), when the lactate that was found to be released from the cells by the initial glucose metabolism had already been taken up and metabolized (Fig. 2b). Subsequently, severe cell toxicity (Fig. 2d, e) and cell detachment (Fig. 2j, m) were observed for these starved astrocytes. For cultures that had been exposed to 5 mM glucose, a high cellular ATP content remained throughout a 3 d incubation period (Fig. 2c). For this condition, no obvious alterations in cell viability (Fig. 2d, e) and the cell morphology (Fig. 2h, k, n) were observed for incubations of up to 72 h.

### Test for the Potential of Extracellular Substrates to Maintain the High Cellular ATP content of Astrocytes During Glucose Deprivation

During a 24 h incubation in glucose-free buffer, the specific ATP content of cultured astrocytes was lowered to around 30% of the initial value (Fig. 2c; Table 1). To test for extracellular substrates that may be able to prevent this decline in cellular ATP levels, astrocytes were incubated for 24 h in glucose-free buffer supplemented with 5 mM of hexoses or substrates that are known to be metabolized by mitochondrial pathways (Table 1). None of the conditions applied compromised the cell viability, as indicated by the absence of any significant increase in extracellular LDH activity (Table 1). The decline in the specific ATP content during incubation in glucose-free buffer for 24 h was completely prevented by supplementation of the buffer with 5 mM glucose, mannose or fructose or with 5 mM of the known astrocytic mitochondrial substrates lactate, pyruvate, β-hydroxybutyrate or acetate (Table 1). In contrast, ATP levels were not maintained, if glucose had been replaced by galactose or 2-deoxyglucose (Table 1).

**Table 1:** **ATP content in glucose-deprived astrocytes after incubation with various extracellular substrates.** Cultured astrocytes were incubated for 24 h in glucose- and amino acid-free incubation buffer in the absence (None) or the presence of 5 mM of the indicated substrates before the specific cellular ATP content and the extracellular LDH activity were determined. The ATP contents are given as nmol/mg and in percent of the initial specific cellular ATP content. 100% ATP content corresponds to 23.5 ± 0.7 nmol/mg. The initial cellular LDH activity of the cultures was 127 ± 31 nmol/(min × well). The data represent mean values ± SD of values obtained in experiments performed on 4 independently prepared cultures. The significance of differences (ANOVA with Bonferroni post hoc test) as compared to the values obtained for the None condition is indicated by *p < 0.05, **p < 0.01 and ***p < 0.001. The significance of differences compared to the data obtained for glucose-treated cells is indicated by ^+^p < 0.05, ^++^p < 0.01 and ^+++^p < 0.001

Substrate	LDH release	ATP content	ATP content
	(% of initial cellular LDH activity)	(nmol/mg)	(% of initial cellular ATP content)
None	7 ± 4	6.5 ± 0.6^+++^	28 ± 3^+++^
Glucose	3 ± 3	21.0 ± 0.9^***^	89 ± 3^***^
Mannose	1 ± 6	22.0 ± 2.4^***^	94 ± 10^***^
Fructose	2 ± 2	25.5 ± 1.2^***, +^	108 ± 5^***, +^
Galactose	4 ± 4	10.6 ± 1.9^+++^	45 ± 8^+++^
2-Deoxyglucose	14 ± 11	7.0 ± 0.9^+++^	30 ± 4^+++^
Lactate	4 ± 3	23.6 ± 2.4^***^	100 ± 10^***^
Pyruvate	2 ± 5	21.5 ± 2.2^***^	91 ± 9^***^
β-Hydroxybutyrate	3 ± 3	20.1 ± 2.2^***^	85 ± 9^***^
Acetate	4 ± 3	19.6 ± 1.5^***^	83 ± 6^***^

### Consequences of a Modulation of Mitochondrial Respiratory Chain Activity on the Astrocytic ATP Content

**Fig. 3:**
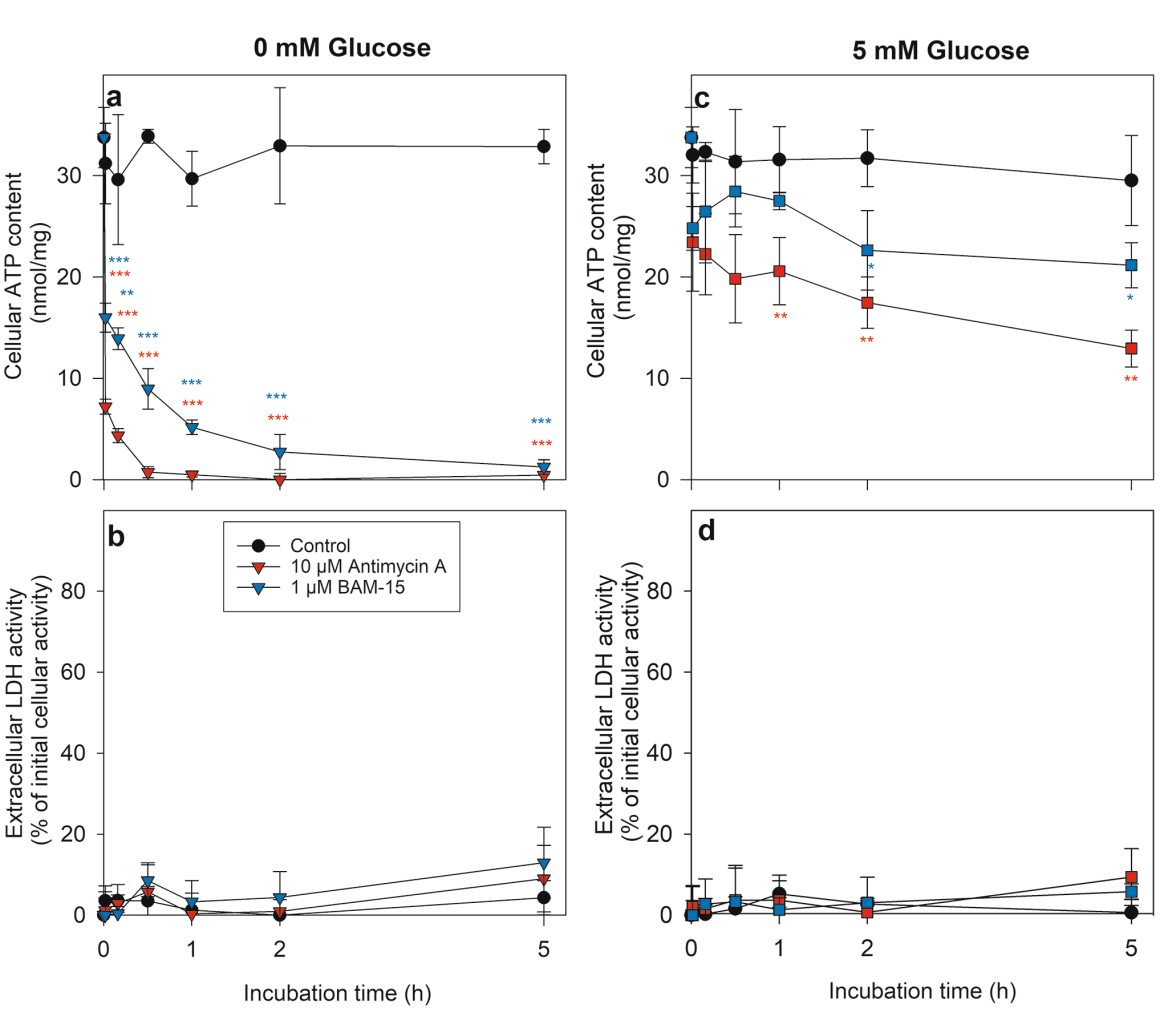
**Effects of antimycin A and BAM-15 on the ATP content of cultured astrocytes.** The cultures were incubated for up to 5 h in glucose-free (a, b) or glucose-containing (5 mM) (c, d) incubation buffer in the absence (control) or the presence of 10 µM antimycin A or 1 µM BAM-15. For the given incubation times the specific cellular ATP content (a, c) and the extracellular LDH activity (b, d) were determined. The cultures had an initial protein content of 130 ± 17 µg/well and an initial cellular LDH activity of 134 ± 22 nmol/(min × well). The data represent mean values ± SD of results obtained in experiments performed on 3 independently prepared cultures. The significance of differences (ANOVA with Bonferroni post hoc test) compared to the data obtained for cells treated in the absence of antimycin A or BAM-15 (control) are indicated in the colours of the respective symbols by * p < 0.05, ** p < 0.01 and *** p < 0.001

As mitochondrial oxidative phosphorylation is known to generate large amounts of cellular ATP, it was investigated whether and how an inhibition or activation of the mitochondrial respiratory chain may affect the observed maintenance of cellular ATP content in glucose-depleted astrocytes. Consistent with results obtained before (Fig. 2c), the high specific cellular ATP content was maintained during an incubation for 5 h in glucose-free buffer (Fig. 3a). In contrast, ATP levels in glucose-deprived astrocytes were quickly depleted within minutes in the presence of the respiratory chain complex III inhibitor antimycin A [30] or the uncoupler BAM-15 [31, 32], lowering the specific cellular ATP content to 1% (antimycin A) and 20% (BAM-15) of the initial value already within 30 min (Fig. 3a).

Application of antimycin A or BAM-15 in the presence of 5 mM glucose lowered also the ATP content of astrocytes. However, these declines were slower and less severe. After an exposure for 10 min to antimycin A or BAM-15, the ATP content of glucose-fed astrocytes accounted for around 70% and 80%, respectively, of the initial ATP level and even after 5 h of incubation these cells still contained around 40% (antimycin A) or 60% (BAM-15) of the ATP content of control cultures (Fig. 3c). None of the applied conditions compromised cell viability during the incubation period of up to 5 h as indicated by the absence of any significant increase in extracellular LDH activity (Fig. 3b, d).

### ATP Levels in Astrocytes After Inhibition of Mitochondrial Uptake of Pyruvate and/or Fatty Acids

**Fig. 4:**
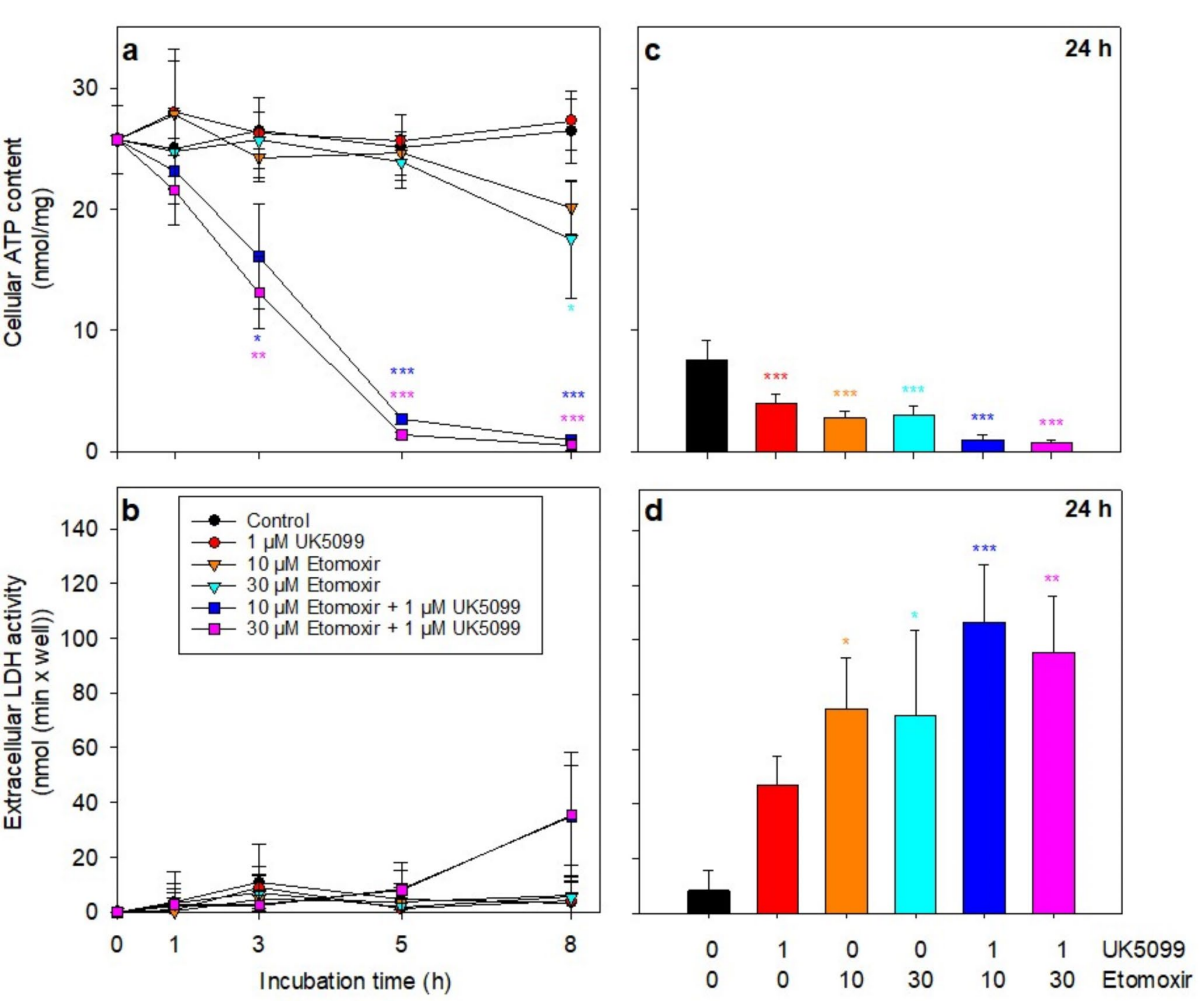
**Effects of an inhibition of mitochondrial uptake of pyruvate and/or fatty acids on the ATP content of cultured astrocytes**. The cells were incubated for up to 8 h (a, b) or for 24 h (c, d) in a glucose- and amino acid-free incubation buffer in the absence (control) or the presence of 1 µM UK5099 and/or 10 or 30 µM etomoxir. For the given incubation times the specific cellular ATP content (a, c) and the extracellular LDH activity (b, d) were determined. The cultures had an initial protein content of 173 ± 13 µg/well and an initial cellular LDH activity of 137 ± 15 nmol/(min × well). The data represent mean values ± SD of results obtained in experiments performed on 3 independently prepared cultures. The significance of differences (ANOVA with Bonferroni post hoc test) compared with the data obtained for cells treated in the absence of inhibitors are indicated in the colours of the respective symbols by * p < 0.05, ** p < 0.01 and *** p < 0.001

The rapid consumption of ATP after inhibition or uncoupling of the respiratory chain demonstrates that mitochondrial ATP generation is essential for maintaining a high cellular ATP content in glucose-depleted astrocytes. To test for a potential consumption of pyruvate or fatty acids as endogenous fuels for mitochondrial oxidation processes to generate ATP, glucose-starved astrocytes were incubated with UK5099, an inhibitor of the mitochondrial pyruvate carrier [21, 33] and/or with etomoxir, an inhibitor of the carnitine palmitoyltransferase 1 [34, 35], in order to prevent the uptake of these potential substrates into mitochondria and their subsequent oxidation to regenerate ATP. In glucose-free buffer the high cellular ATP content of astrocytes was maintained for up to 8 h (Fig. 4a), while ATP levels declined during a 24 h incubation to around 30% of the initial content (Fig. 4b), as shown earlier (Fig. 2; Table 1).

In the presence of 1 µM UK5099 the ATP content of the cells (Fig. 4a) and the cell viability (Fig. 4c) remained high for at least 8 h, but were significantly lower than that of control cells after 24 h of incubation (Fig. 4b). The cell viability was also found to be severely compromised after 24 h (Fig. 4d). Similarly, an application of 10 µM or 30 µM etomoxir alone had little effect on the cellular ATP content (Fig. 4a) and the cell viability (Fig. 4b) during the initial 5 h of incubation. ATP levels were found to be slightly declined after 8 h of incubation (Fig. 4a), although those alterations did not reach the level of significance. In contrast, ATP levels below 10% of that of control cells (Fig. 4c) and severe cell toxicity (Fig. 4d) were observed for astrocytes that had been incubated for 24 h with etomoxir. Although the individual inhibitors did not affect astrocytic ATP content within a 5 h incubation, a co-application of UK5099 plus etomoxir depleted the cellular ATP contents in a time-dependent manner almost completely within 5 h (Fig. 4a), although the viability of the cells was not compromised during this time period (Fig. 4b). Longer incubations (24 h) with UK5099 plus etomoxir caused both severe cell toxicity (Fig. 4d) and an almost complete loss of cellular ATP (Fig. 4c). As hardly any difference was observed for the consequences of an exposure of astrocytes with UK5099 plus etomoxir in concentrations of 10 µM and 30 µM (Fig. 4), it is assumed that the presence of 30 µM etomoxir was sufficient to efficiently inhibit the mitochondrial uptake of activated long-chain fatty acids in astrocytes under the conditions used.

## Discussion

To investigate how glucose deprivation, replacement of glucose by other metabolic substrates as well as an application of modulators of mitochondrial transport processes and metabolism affect cellular ATP levels in astrocytes, we have used confluent astrocyte cultures of an age between 14 and 28 days. The average specific ATP content of untreated cultures was calculated to be 27.9 ± 4.7 nmol/mg. This specific ATP content is in the range between 19 and 40 nmol/mg that has been reported previously for astrocyte cultures by many groups [18, 19, 21, 36, 37]. Assuming that most of the ATP determined in astrocytic lysates represent cytosolic ATP, the ATP concentration in the cytosol would account to around 7 mM, as calculated by using a cytosolic volume of 4.1 µL/mg for cultured rat astrocytes [38]. Such a high cytosolic concentration appears not to be essential for efficiently driving ATP-mediated reactions, as the K_M_ values for ATP of kinases and ATP-dependent transporters, such as hexokinase [39], creatine kinase [40] or the Na^+^-K^+^-ATPase [41] are usually in the concentration range below 1 mM. However, considering the large number of kinases and ATP-dependent transporters in astrocytes as well as the known high demand of astrocytic Na^+^-K^+^-ATPase for ATP [42], a high ATP concentration may be needed as a short time buffer to maintain ATP-dependent reactions under conditions of a fluctuating ATP regeneration.

Astrocytes are known to efficiently metabolize glucose and to produce ATP via glycolysis [8]. However, even after glucose depletion the high cellular ATP content was maintained for up to 8 h, consistent with literature data [43], demonstrating that astrocytes have sufficient metabolic reserve capacity for hours to compensate for a lack of exogenous glucose under the conditions used. However, after 24 h of glucose-deprivation the specific cellular ATP content was lowered to around 30% of the initial ATP content without any obvious impairment of cell viability. LDH release from damaged cells and altered cell morphology were only observed after longer starvation. Similarly, a loss in cellular ATP content to around 30% that was followed by delayed cell death was found for astrocytes that had initially been exposed to 1 mM glucose after all glucose-derived lactate in the medium had been consumed. Thus, the viability of astrocytes appears only to be compromised, if cellular ATP levels are lowered to values below 30% of the normal high ATP content. This finding is consistent with the reported threshold level of around 25% of the normal ATP content that is crucial to keep cultured astrocytes alive [44] and suggests that a further decline of ATP content below 25% that is connected to cell death is the consequence of an exhaustion of the endogenous stores that provide the substrates to regenerate ATP.

The loss in cellular ATP contents in glucose-deprived astrocytes during a 24 h incubation was prevented in presence of mannose or fructose, but not by galactose, as expected from the known potential of cultured astrocytes to metabolize such hexoses [45–47]. In contrast, 2-deoxyglucose, a hexose which is taken up and phosphorylated in astrocytes [48] but cannot be further metabolized in glycolysis, was - as expected - unable to help preventing the cellular ATP loss in glucose-deprived astrocytes. However, mitochondrial substrates such as lactate, pyruvate, acetate or β-hydroxybutyrate, which are known to be consumed by cultured astrocytes [5, 21], were able to fully maintain the high ATP content in glucose-depleted astrocytes for at least 24 h. This demonstrates that the oxidation of mitochondrial substrates and mitochondrial oxidative phosphorylation can fully compensate for an impaired glycolytic ATP production in cultured astrocytes to maintain a high cellular ATP concentration.

Impairment of mitochondrial respiration caused a rapid depletion of cellular ATP contents in glucose-deprived astrocytes. This finding is consistent with literature data that report the need to simultaneously impair glycolysis and mitochondrial processes to deplete astrocytes of their ATP [18–21]. In contrast, an inhibition of the respiratory chain by antimycin A or uncoupling by BAM-15 in the presence of glucose lowered the ATP content of astrocytes only partially within hours, consistent with reported data for other mitochondrial inhibitors [19, 36]. Thus, although antimycin A [16, 49] and BAM-15 (unpublished data) doubled glycolytic lactate production in cultured astrocytes, this stimulation of glycolysis appears to be unable to fully compensate for an impaired mitochondrial ATP regeneration. This can be explained by the much larger yield of ATP that is obtained by oxidation of glucose-derived pyruvate in mitochondria compared to the ATP that can be generated from glucose during glycolysis.

The rapid decline in cellular ATP levels of glucose-deprived astrocytes in presence of antimycin A or BAM-15 clearly demonstrates that mitochondrial ATP regeneration is essential for maintaining a high cellular ATP content during glucose-deprivation. Considering the ability of astrocytes to maintain ATP for many hours, substantial amounts of intracellular sources have to be available in astrocytes to provide the substrates for mitochondrial ATP regeneration under the conditions used. One intracellular source that may help to maintain the cellular ATP concentration on a high level is the polysaccharide glycogen. Glycogen contents in cultured astrocytes account for around 80 nmol glycosyl residues/mg protein and this glycogen is rapidly mobilized after glucose-depletion with a halftime of 7 min [50]. Subsequently, glycogen-derived glucose-6-phosphate is glycolytically metabolized to pyruvate that could be further oxidized in mitochondria, but has been reported to be mainly reduced to lactate that is released from these astrocytes [51]. Nevertheless, as extracellular lactate is consumed even in low concentrations by glucose-depleted astrocytes [16, 21], this lactate as well as lactate derived from free glucose present in the cells at the onset of the incubation [52] or pyruvate from other metabolic processes may serve as substrate for mitochondrial ATP regeneration. Other potential substrates for mitochondrial ATP regeneration in astrocytes may be fatty acids as astrocytes possess lipid droplets that contain substantial amounts of long-chain fatty acids [1, 13].

To test for an involvement of pyruvate and/or fatty acid oxidation in the mitochondrial ATP regeneration in glucose-deprived astrocytes, we applied UK5099, an inhibitor of the mitochondrial pyruvate carrier that already in a concentration of 1 µM efficiently prevents mitochondrial pyruvate consumption by astrocytes [21] and etomoxir, an inhibitor of carnitine palmitoyltransferase I, which is essential for the uptake of activated long-chain fatty acids from the cytosol into mitochondria for subsequent β-oxidation [34, 35]. Interestingly, none of these inhibitors alone affected the high cellular ATP content for many hours, suggesting that more than one process is responsible to provide metabolic fuels for mitochondrial ATP regeneration. Indeed, co-application of both inhibitors abolished cellular ATP contents within 5 h. Thus, the mitochondrial uptake and metabolism of both pyruvate and fatty acids are likely to strongly contribute to the maintenance of a high ATP level in glucose-deprived astrocytes. Both processes appear to provide alone already sufficient fuels for ATP synthesis for several hours, thereby having even the capacity to compensate for an impairment of the other process. However, each of the cellular sources that fuel substrates into mitochondrial pathways in starved astrocytes via pyruvate and/or activated fatty acids appears to be exhausted during a 24 h glucose-deprivation as demonstrated by the lowered cellular ATP contents and the high toxicity observed for the inhibitor-treated astrocytes.

In conclusion, glucose-depleted astrocytes maintain a high initial ATP content for many hours by mitochondrial ATP regeneration that is mainly fueled by oxidation of pyruvate and fatty acids, which are likely to be provided from endogenous stores such as glycogen and lipid droplets. In contrast, accelerated glycolysis in glucose-treated astrocytes after impairment of mitochondrial respiration can only partially maintain the high ATP content. Further studies are now required to investigate in more detail the intracellular processes that provide pyruvate and fatty acids for mitochondrial ATP synthesis in astrocytes, the potential use of extracellular amino acids and fatty acids as fuels for cellular ATP synthesis and how ATP consumption can be affected by modulation of the main consumers of cellular ATP.
